# Efficacy of estriol in inhibiting epithelial proliferation in mammary fibroadenoma: randomized clinical trial

**DOI:** 10.1590/S1516-31802007000600008

**Published:** 2007-11-01

**Authors:** Rodrigo Augusto Fernandes Estevão, Edmund Chada Baracat, Ângela Flávia Logullo, Celina Tizuko Fujiyama Oshima, Afonso Celso Pinto Nazário

**Affiliations:** Mastology Sector of Department of Gynecology and Department of Pathology, Universidade Federal de São Paulo — Escola Paulista de Medicina (Unifesp-EPM), São Paulo, Brazil

**Keywords:** Fibroadenoma, Contraceptives oral, Estriol, Ki-67 antigen, Myc genes, Fibroadenoma, Anticoncepcionais orais, Estriol, Antígeno Ki-67, Genes myc

## Abstract

**CONTEXT AND OBJECTIVE:**

Mammary fibroadenoma is a disease that affects a large number of women of reproductive age. The aim of this study was to evaluate the proliferative activity of mammary fibroadenoma through expression of Ki-67 and c-myc antigens, following administration of oral contraceptive with or without estriol

**DESIGN AND SETTING:**

Placebo-controlled double-blind randomized clinical trial in the Mastology Sector of the Department of Gynecology, Universidade Federal de São Paulo

**METHODS:**

Thirty-three fibroadenoma patients were studied. Ten women (group 1) took an oral contraceptive constituted by levonorgestrel and ethinyl estradiol together with placebo manufactured in the same capsule for four consecutive cycles with a seven-day interval between them. The other 23 patients (group 2) took the same oral contraceptive together with estriol, which was put into the same capsule and used in the same way as among the group 1 patients. After four cycles, the nodules were surgically removed and sent for immunohistochemical analysis of Ki-67 and c-myc expression

**RESULTS:**

The Ki-67 and c-myc analysis did not reveal any significant differences between the study groups. The values were 9.16 and 10.54 for group 1 and 10.86 and 17.03 for group 2, respectively. There was a tendency towards higher expression of antigens in group 2

**CONCLUSION:**

Our results showed that there was no significant statistical difference in Ki-67 and c-myc expression between our study groups, but only a tendency towards higher expression among users of oral contraceptives containing estriol

## RESUMEN

**CONTEXTO E OBJETIVO:**

O fibroadenoma mamário é uma doença que atinge um grande número de mulheres na idade reprodutiva. O objetivo foi avaliar a atividade proliferativa do fibroadenoma mamário, através da expressão do Ki-67 e do c-myc, após a administração de anticoncepcional oral, associado ou não ao estriol.

**TIPO DE ESTUDO E LOCAL:**

Ensaio clínico randomizado, duplo-cego, placebo controlado, realizado na Universidade Federal de São Paulo a nível terciário.

**MÉTODOS:**

Foram estudadas 33 pacientes portadoras de fibroadenoma, atendidas no setor de Mastologia da Disciplina de Ginecologia da Universidade Federal de São Paulo — Escola Paulista de Medicina (Unifesp-EPM), sendo que 10 mulheres constituíram o grupo 1, e utilizaram anticoncepcional oral composto de levonorgestrel e etinilestradiol, associados a placebo na mesma cápsula por quatro ciclos consecutivos, com intervalo de sete dias entre cada um. As restantes 23 pacientes alocaram-se no grupo 2 e ingeriram, além do anticoncepcional oral descrito acima, um comprimido de estriol, que foi manufaturado na mesma cápsula e foi utilizado da mesma forma que nas pacientes do grupo 1. Ao final dos quatro ciclos, praticou-se a exérese cirúrgica dos nódulos, com posterior envio para análise imunoistoquímica de Ki-67 e c-myc.

**RESULTADOS:**

A análise com Ki-67 e c-myc não revelou diferença significante entre os grupos estudados, que foi de 9,16 e 10,54 no grupo 1 e de 10,86 e 17,03 no grupo 2, respectivamente, apesar de ter havido tendência a maior expressão dos marcadores entre as pacientes do grupo 2.

**CONCLUSÃO:**

Nossos resultados demonstram não haver diferença estatisticamente significante na expressão de Ki-67 e de c-myc entre os grupos em estudo, apenas uma tendência a sua maior expressão entre as usuárias de anticoncepcional e estriol.

## INTRODUCTION

Fibroadenoma is the most common benign tumor of the female breast. It is a neoplasm formed by glandular and fibrous tissues and occurs in young patients aged 20 to 30 years. It is the most common benign disease among females under the age of 35 years and it occurs without symptoms in 25% of the cases. It is multiple in 13 to 20%^1-3^ and is more common among blacks than among whites.^4-6^

Fibroadenomas grow as well-limited mobile spherical nodules and do not become attached to neighboring breast tissue. They are more frequently located in the left breast and in the upper external quadrant. Their size ranges from small lesions of under 1 cm to huge dimensions, such as 10 to 15 centimeters (giant fibroadenoma).^7^

Phyllodes tumor, fibrosarcoma, asymmetric juvenile giant breast, mammary cysts, galactocele, hamartoma, adipose necrosis, hematoma and breast cancer are the possible differential diagnoses.^1^

There are many different types of treatment for breast fibroadenoma. Tumors of less than one centimeter in diameter may undergo partial or total regression in up to one third of the cases.^8^ Wait-and-see management may be the best option, particularly in the cases of small tumors in females under the age of 25 years or under the age of 35 years without familial risk of breast cancer.^9^ In other cases, surgical extraction may be considered.

Estrogens seemed to be linked to fibroadenoma genesis.^10^ Fibroadenoma occurs mainly in young females, who frequently choose oral contraceptives as their contraceptive method.

Oral contraceptives contain synthetic estrogens and progestogens in their formulae. The estrogen most used in oral contraceptives is ethinyl estradiol. The high doses used in the past have today come down to 15 or 30 µg/pill.^11^ The progestogen component varies greatly between different synthetic compounds and acts like a natural progesterone. Oral contraceptives are thus differentiated by their power to reproduce progestogenic effects.^12^

Inhibition of fibroadenoma growth using oral contraceptives or hormone replacement therapy is still a controversial topic. While Ravnihar et al.^13^ demonstrated that hormone therapy is a protection factor, Sitruk-Ware et al.^14^ did not observe such influence.

The greatest controversy relating to hormonal oral contraception is its association with the risk of developing breast cancer. Epidemiological studies show conflicting conclusions and it is important to emphasize that, because large numbers of people use oral contraception, even a small rise in risk may represent a large number of cancer cases. The Cancer and Steroid Hormone (CASH) study,^15^ published in 1986, did not show any association between hormonal oral contraception and breast cancer. Marchbanks et al.^16^ conducted a case-control study among women aged 35 to 40 years, and found a relative risk of 1.0 for women who were taking oral contraceptives and 0.9 for those who had already taken them.

Knowledge of cellular theory in pathology has clearly shown that cell behavior is related to nuclear characteristics. This knowledge has evolved such that it has been found that the nucleus is the locus of the genetic information that will be transmitted through cells.^17^

Simomoto et al.^18^ studied the epithelial kinetics of fibroadenomas during menstrual cycle by morphometric analysis. The mitotic index and average nuclear volume did not show significant differences between phases, which therefore refutes the possibility of fibroadenoma epithelial cyclicity as occurs in normal breasts. Because of the strange proliferation of lobular epithelium that they observed, they suggested that fibroadenoma formation was modulated by paracrine mechanisms.

Hueb^19^ and Taniguchi^20^ compared the cellular proliferative nuclear antigen (CPNA) levels in epithelium with fibroadenoma, among users and non-users of oral contraceptives. They did not find any significant differences, and only a tendency towards higher expression among non-users.

The results from these last studies suggested that oral contraceptives did not inhibit the proliferative activity of epithelium with fibroadenoma. It would therefore be of interest to evaluate whether some substance, used concomitantly with oral contraceptives, might inhibit the cellular kinetics of this tumor. Thus, we raised the hypothesis that estriol might block the oral contraceptive effect on fibroadenoma.

Estriol is, in fact, the peripheral metabolite of estrone and estradiol, rather than a secretory product from the ovary. Its production occurs because of metabolic detoxification, i.e. conversion of active substances into less active forms.^21^

In reviewing the literature, we were surprised to find that there was no published research evaluating the effect of estriol, with or without oral contraceptives, on fibroadenomas and, in particular, on its proliferative activity.

This proliferative activity may be evaluated by several techniques such as mitosis counting; immunohistochemical analysis of CPNA, Ki-67 and c-myc; and DNA content evaluation by means of tritiated thymidine and flux cytometry.^22^ Identification of these nuclear antigens by immunohistochemical analysis, thereby showing their presence in cells that are undergoing a proliferation process and absence in cells that are at rest, enables measurement of the proliferation of a given tissue.

Among these antigens is Ki-67, which was first described by Gerdes et al.^23^ This is detected by means of the monoclonal antibody for Ki-67, which is in immunoglobulin class G1 (IgG1). This reacts with a nuclear antigen expressed in all the phases of the cellular cycle. It is obtained following immunization of mice with the nuclear extract from Hodgkin's lymphoma of cellular lineage L428, and its name is derived from the fact that its original clone in the culture plate was in the 67^th^ position.^24^

Although the exact function of the Ki-67 antigen remains unknown, it is widely utilized in pathology to evaluate the growth fraction of normal and neoplastic tissues, particularly in studies implicating its possible prognostic value. In solid tumors, an association between high proliferation rate and aggressiveness has been proven.

C-myc was first described as a retroviral oncogene in chickens (myelocytomatosis virus) by Bishop^25^ in 1982. Today, it has a central role in diagnosing malignant proliferation and transformation of human and animal cells.^26^ It was discovered as a transforming sequence in the avian retrovirus MC29^27^ and was subsequently identified in the vertebrate genome. The human c-myc oncogene is located distally in the long arm of chromosome 8, in the 8q24 region.^28^ Most human malignant tumors show amplification or overexpression of this gene, according to several studies.^29^ It is now known that the c-myc gene plays a part in several cell functions such as replication, growth, metabolism, differentiation and apoptosis.^30^

In physiological situations, the role of c-myc is to promote cell replication in response to extracellular signs, thereby leading quiescent cells to enter the cellular cycle through activation of regulatory target gene transcription of the cellular cycle, such as D1 and D2 cyclins.^31^

Therefore, we postulated that c-myc could be a nuclear protein acting like a transcription factor and contributing at all stages of the carcinogenic process, thereby transforming normal cells into more malignant cells.^32^ Hence, positive findings in immunohistochemical assays might reflect poor prognosis. However, high levels of c-myc in tumors that have already reached certain dimensions could induce apoptosis or increase the sensitivity to certain apoptotic factors, such as cyclins. There is also experimental evidence that it could be an important transcription repressor, thereby obstructing the survival of the tumor in a hostile environment.^33^

## OBJECTIVE

Because of the small number of studies on the real effects of oral contraceptives on breast fibroadenomas, we proposed to evaluate the cellular proliferation of this epithelium tissue according to its expression of cellular proliferation proteins (Ki-67 and c-myc) among users of oral contraceptives, with or without association with estriol, in order to investigate their effects on these tumors.

## METHODS

We started with 70 women who were attending consultations at the outpatient clinic for benign mammary disorders of the Department of Gynecology of Universidade Federal de São Paulo, between March 2001 and January 2005. This sample was obtained through statistical analysis, and consisted only of cases that were referred to our clinic.

The patients were selected according to certain criteria. The inclusion criteria were that the subjects needed to be healthy women with benign mammary tumors diagnosed by clinical, cytological and radiological evaluations, with ages ranging from 19 to 35 years. They needed to have had regular menstruation during the preceding six months, with the last menstrual period known and with normal gynecological and colposcopic examinations.

We excluded patients who had endocrinopathies, who were pregnant or in puerperium, or who presented suspicions of any carcinoma.

This project was analyzed and approved by the Research Ethics Committee of Universidade Federal de São Paulo.

All the patients were evaluated by means of clinical and supplementary examinations such as anamnesis, physical examination, fine-needle aspiration punch, oncological cytology, ultrasound and, when indicated, mammography.

We were only able to make complete observations on 33 patients, and this may have adversely affected our results, since this caused a decrease in the statistical power of the study, considering that a total of 37 patients were lost. Among the reasons for these losses over the course of the study period were the number of consultation that the patients needed to attend in order to reach an indication for surgery;^3^ their lack of resources for coming to our clinic; and their lack of motivation caused by the small benefit attained through the use of medication.

The patients were randomly divided into two groups, which were made up as follows. Group 1 (control) consisted of 10 women with delineated fibroadenoma, who used oral contraceptive constituted by levonorgestrel (0.15 mg) and ethinyl estradiol (0.03 mg) together with placebo inside the same capsule, for up to four consecutive cycles with seven-day intervals between them. Group 2 consisted of 23 patients who also had benign breast tumors and who took the same oral contraceptive, but with 2 mg of estriol instead of the placebo, manufactured in the same capsule, for up to four consecutive cycles. The randomization into these two groups for subsequent analysis was done by the pharmacists who manufactured the medication.

In all cases, we measured the serum progesterone by an immunoassay technique on the day of the biopsy, in order to prove that the oral contraceptive was having an anovulatory effect.

The study was conducted in a randomized double-blind manner. This explains the differences in patient numbers in the two groups used in this study, since the divisions were only known after all the material was collected, i.e. after the tumor biopsies and during the statistical analysis.

The samples obtained from the surgical procedures were subjected to histopathological and immunohistochemical procedures.

### Histopathological method

The samples collected were fixed in 10% buffered formol and then dehydrated in ethyl alcohol, diaphanized in xylol and embedded in paraffin blocks. We made serial thin sections by means of microtomy and then made these up into slides. These slides were sent for immunohistochemical analysis in order to evaluate the Ki-67 and c-myc expression.

### Immunohistochemical method

We mounted sections of 3 µm in thickness on slides and left them to dry in an oven for 12 hours. They were then deparaffinized with subsequent hydration.

The antigen recovery for c-myc antibodies was carried out in a microwave oven, using pH 6.0 citrate buffer. For Ki-67 antibodies, the antigen recovery was carried out in a pressure cooker, also using pH 6.0 citrate buffer.

After washing in water, we performed endogenous peroxidase blockage and incubation with mouse anti-human Ki-67 monoclonal antibodies (MIB-1 clone, Dako, United States) at a titer of 1:100, and with mouse anti-human c-myc (Dako, United States), at a titer of 1:100, in 1% bovine serum albumin (BSA) solution in phosphate-buffered saline (PBS), in a damp chamber at 4° C, for 16 to 18 hours

The development was done by means of a chromogenic substrate (3,3’-diaminobenzidine; D5637, Sigma, United States) with oxygenated water in pH 7.4 PBS. Following this, cover slips were mounted on the slides using Entellan resin (Sigma)

The slides were taken to be positive for the expression of Ki-67 and c-myc if a brown coloration appeared in the cell nuclei

### Interpretation of the results

The monoclonal antibody Ki-67 reacts with nuclear antigens that are present in cells with proliferative activity, thereby showing nuclear positivity. This reaches its peak in the G2 and M phases of the cellular cycle. The antigen expression is negative in quiescent cells in the G0 and early G1 phases.^34^ On the other hand, c-myc promotes cell replication in response to extracellular signs, thereby leading quiescent cells to enter the cellular cycle through activation of target transcription genes.^26^

The cell proliferation index for Ki-67 in epithelium was determined by the ratio (as a percentage) between the total number of stained nuclei and the total number of nuclei counted. The c-myc index was calculated in the same way.

Quantitative image analysis was performed using the Image-Pro Plus software, version 3.0. The images were generated from an Olympus microscope coupled to a Sony CMA-D2 camera.

### Statistical method

The means of the quantitative variables in the two groups were compared using Student's t test for two independent samples, after applying Levene's variance equality test.^34^ The measurements made in the two groups were also compared using the non-parametric Mann-Whitney test.^35^

We took a significance level of 5% to draw conclusions from all the tests performed.

## RESULTS

The two groups were homogeneous with regard to the variables that might act on the cellular kinetics of fibroadenomas (age, pregnancies, parity and abortions), as shown in Table 1.

**Table 1:** Epidemiological data on the patients studied, according to group: 1 (placebo plus oral contraceptive) or 2 (oral contraceptive plus estriol)

Patients	Age (years)	Age at menarche (years)	Number of gestations	Parity	Number of abortions	Lactation (months)
**Group 1**						
IS	25	14	0	0	0	0
RAC	17	11	0	0	0	0
EAP	18	13	0	0	0	0
MCN	22	11	1	1	0	0
PCJ	29	11	0	0	0	0
VCAJ	18	13	0	0	0	0
VSN	19	13	0	0	0	0
MPSS	20	12	0	0	0	0
ACS	23	11	0	0	0	0
IVSS	18	12	0	0	0	0
**Mean**	20.9	12.1				
**Group 2**						
MMC	29	12	0	0	0	0
PPS	20	10	0	0	0	0
CAS	25	13	1	1	0	30
LSA	23	12	0	0	0	0
ACCM	15	11	1	0	1	0
MAL	20	14	0	0	0	0
RNS	34	16	3	3	0	36
RPP	33	15	0	0	0	0
SRA	21	13	0	0	0	0
ROR	18	12	0	0	0	0
FLR	24	13	5	5	0	0
RGLN	26	13	1	1	0	48
HSZ	18	13	0	0	0	0
SMS	18	13	0	0	0	0
NRG	14	9	0	0	0	0
SSS	30	12	3	2	1	26
RALS	30	10	2	2	0	24
SLOC	16	12	0	0	0	0
RMN	17	12	0	0	0	0
DT	24	13	1	0	1	0
JBS	23	15	0	0	0	0
RMM	29	13	0	0	0	0
JMPS	20	15	0	0	0	0
**Mean**	22.9	12.7				

Tables 2 to 5 show the percentages of stained nuclei of epithelial cells in investigating Ki-67 and c-myc antigens, in relation to the total number of nuclei analyzed, for all patients in the study. We also present the means and standard errors for the measurements in groups 1 and 2.

**Table 2:** Number of positive and negative epithelial cell nuclei and total number of nuclei analyzed in fibroadenoma, in immunohistochemical analysis for Ki-67 antigen in group 1 (oral contraceptive plus placebo)

Patients	Positive nuclei	Negative nuclei	Total number of nuclei analyzed	Percentage (%)
VCAJ	1231	3053	4284	28.73
IVSS	641	3313	3954	16.21
ACS	445	3072	3517	12.65
VSN	431	3637	4068	10.59
MPSS	212	2944	3156	6.72
RAC	186	2692	2878	6.46
MCN	164	2486	2650	6.19
EAP	67	3481	3548	1.89
PCJ	53	3128	3181	1.67
IS	16	3205	3221	0.50
**Mean**				9.16
**Standard error**				2.69

**Table 3:** Number of positive and negative epithelial cell nuclei and total number of nuclei analyzed in fibroadenoma, in immunohistochemical analysis for c-myc antigen in group 1 (oral contraceptive plus placebo)

Patients	Positive nuclei	Negative nuclei	Total number of nuclei analyzed	Percentage (%)
IS	1681	2713	4394	38.26
MCN	1395	3180	4575	30.49
ACS	480	2882	3362	14.28
VSN	485	4434	4919	9.86
VCAJ	159	2525	2684	5.92
PCJ	159	3454	3613	4.40
IVSS	60	2333	2393	2.51
MPSS	57	3544	3601	1.58
EAP	13	1999	2012	0.65
RAC	17	2660	2677	0.64
**Mean**				10.86
**Standard error**				4.19

**Table 4:** Number of positive and negative epithelial cell nuclei and total number of nuclei analyzed in fibroadenoma, in immunohistochemical analysis for Ki-67 antigen in group 2 (oral contraceptive plus estriol)

Patients	Positive nuclei	Negative nuclei	Total number of nuclei analyzed	Percentage (%)
RPP	1287	4118	5405	23.81
MAL	1103	3738	4841	22.78
HSZ	909	4308	5217	17.42
ROR	608	3077	3685	16.50
NRG	661	3680	4341	15.23
MMC	623	3517	4140	15.05
ACCM	336	1972	2308	14.56
RMN	661	4141	4802	13.77
PPS	537	3376	3913	13.72
SRA	449	2908	3357	13.38
SLOC	397	2695	3092	12.84
RMM	653	4638	5291	12.34
SSS	495	3579	4074	12.15
CAS	297	2994	3291	9.02
DT	238	2719	2957	8.05
RGLN	232	3293	3525	6.58
LSA	140	3205	3345	4.19
JBS	136	3829	3965	3.43
FLR	95	3357	3452	2.75
RALS	98	3819	3917	2.50
SMS	38	3903	3941	0.96
JMPS	30	3157	3187	0.94
RNS	19	4497	4516	0.42
**Mean**				10.54
**Standard error**				1.43

**Table 5:** Number of positive and negative epithelial cell nuclei and total number of nuclei analyzed in fibroadenoma, in immunohistochemical analysis for c-myc antigen in group 2 (oral contraceptive plus estriol)

Patients	Positive nuclei	Negative nuclei	Total number of nuclei analyzed	Percentage (%)
SSS	1262	1765	3027	41.69
RNS	1681	2427	4108	40.92
JMPS	1452	2460	3912	37.12
PPS	1026	1872	2898	35.40
SLOC	855	1600	2455	34.83
CAS	1674	3209	4883	34.28
MAL	1023	2231	3254	31.44
RPP	877	2556	3433	25.55
RGLN	810	3201	4011	20.19
FLR	517	2586	3103	16.66
DT	573	3679	4252	13.48
SMS	647	4230	4877	13.27
MMC	354	2514	2868	12.34
NRG	399	3486	3885	10.27
ACCM	252	2664	2916	8.64
RMN	177	4086	4263	4.15
HSZ	100	3133	3233	3.09
RMM	94	4385	4479	2.10
LSA	50	2696	2746	1.82
ROR	48	2863	2911	1.65
SRA	39	2723	2762	1.41
JBS	34	3824	3858	0.88
RALS	12	2717	2729	0.44
**Mean**				17.03
**Standard error**				3.09

OC = oral contraceptives; E3 = estriol; SGF = stromal growth factors; EGF = epithelial growth factors; SIGF = stromal inhibitory growth factors.

Table 6 indicates that there was no statistically significant difference in fibroadenoma between the ratios of stained cell nuclei to total number of nuclei analyzed in the investigation of Ki-67 and c-myc antigens in the two groups. This was proven by Student's t-test and the Mann-Whitney test.

**Table 6:** Means and standard errors for nuclei positive for Ki-67 and c-myc in the two groups

Antigens	Group 1 OC + Placebo		Group 2 OC + Estriol		Descriptive level (p)	
	Mean	Standard error	Mean	Standard error	Student's t-test	Mann-Whitney
Ki-67	9.161	2.694	10.858	4.189	0.625	0.389
c-myc	10.539	1.429	17.027	3.086	2.666	0.256

OC = oral contraceptive.

## DISCUSSION

Fibroadenoma is a mixed neoplasm containing variable quantities of epithelial and conjunctive tissue that has great importance, because it occurs during the menacme. Some authors have considered that fibroadenomas are a localized form of nodular hyperplasia of the stroma and glandular component, i.e. a variety of fibrocystic disease known as adenomatous hyperplasia.^36^ However, this condition is diffuse and does not present the typically nodular shapes of fibroadenomas.^37^

Sawhney et al^24^ studied fibroadenomas and phyllodes tumors and showed that a special relationship exists between the mitosis in stroma and epithelial tissue concentrations. They used a computerized morphometric model and quantified the distribution of the epithelium in a sequence of concentric rings surrounding the mitoses of the fibroblasts. They observed that, when the distance between stroma and epithelium was greater than 200 µm, the stromal mitotic activity was expendable and was limited to this reach of action. This distance corresponds to the passive diffusion reach of oxygen, which therefore leads to the hypothesis that control over fibroadenoma proliferation depends mainly on local humoral factors of a paracrine nature, rather than endocrine mechanisms. Thus, in stromal regions where the proliferative activity is higher, they found greater epithelium concentration, whereas in stromal regions where the epithelial tissue was distant, the fibroblast mitotic activity was lower

Hasebe et al.^38^ used analysis of CPNA expression to study the patterns of fibroadenomas with low stromal activity, hypercellular fibroadenomas with higher stromal activity and phyllodes tumors, in which the stromal activity was greatest. They found low expression of CPNA in fibroblasts from standard fibroadenomas and that, as the stromal cellularity increased, such as in hypercellular fibroadenoma and phyllodes tumors, there was an increase in CPNA expression. This expression was greatest in the phyllodes tumors. Therefore, they suggested that stromal cellularity was regulated by the expression of fibroblast growth factors and by their receptors in paracrine growth pathways. Thus, fibroadenoma growth would depend on stromal compartment proliferation, induced by growth factors produced in the epithelium.

In fibroepithelial tumors, the epithelial elements are inside a kind of stroma with an abnormal degree of proliferation but with a uniform pattern. If stromal mitotic activity due to paracrine mechanisms depended on humoral factors produced by epithelium, we would expect that the proliferative activity would be higher the closer the epithelium was, which is what Sawhney et al.^24^ observed. These authors recognized that the epithelium had the capacity to produce platelet-derived growth factor (PDGF), epidermal growth factor (EGF) and type 1 insulinoid growth factor (IGF-1), which act on fibroblasts to stimulate DNA synthesis and induce their growth.

These findings made authors like Sawhney et al.^24^ and Pasqualini et al.^39^ accept the presence of local loop control, in which growth factors produced in epithelium act in the stroma, thereby making it proliferate. This proliferated stroma will produce new growth factors that are necessary for epithelial growth and thus increase the quantity of fibroadenoma. This interdependence is lost in malignant neoplasms, but it is still preserved in fibroepithelial tumors that are specialized lesions from breast stroma with the capacity to stimulate growth of the neighboring epithelium, thereby reaching an equilibrium between growth and inhibition factors. In this way, the slow growth of fibroadenomas and their stabilization after reaching a given size in a great number of patients can be explained.^7^ In small numbers of patients, this link is lost and sarcomatous transformation may take place when the epithelium does not inhibit stromal growth, or carcinomatous transformation when the stroma does not block epithelial proliferation (by inhibiting growth factors).

The immunohistochemical expression of Ki-67 antibodies (MIB-1) was studied by Umekita and Yoshida.^40^ This protein is also related to the cellular cycle and to the proliferation rate in fibroadenomas and phyllodes tumors. They tried to make a correlation between the malignancy power of these tumors and discovered that increases in the MIB-1 stromal index were related to increases in the degree of malignancy, but that the expression of the antibody in epithelium was the same for all kinds of fibroadenomas. They also observed that the stromal expression of MIB-1 in hypercellular varieties was 2.5 times greater than in standard fibroadenoma. In relation to phyllodes tumors, they demonstrated that there was lower expression of the antibodies in epithelium and higher expression in stroma as the degree of malignancy increased, thus showing the importance of stromal activity for increasing the size of these tumors.

The estrogen component of the oral contraceptives used in our study (ethinyl estradiol) is known to be 100 to 200 times stronger than estradiol, which is the estrogen generally found in circulation.^41^ Thus, ethinyl estradiol stimulates the epithelial compartment more strongly than estradiol does, and this action takes place through mediation by the estrogen receptors, which have greater presence in fibroadenoma than in normal tissue.^36^

The greater action of ethinyl estradiol on epithelium leads it to proliferate, thus giving rise to greater expression of proteins relating to the cellular cycle, such as the Ki-67 and c-myc used in our study. It has also been found that, through paracrine control, higher production of stromal growth inhibitory factors may induce lower stroma proliferation. The final effect is a decrease in fibroadenoma dimensions, considering that the stromal compartment is responsible for fibroadenoma size.^42^ This theory was corroborated by our findings, in which we observed lower expression of Ki-67 and c-myc (9.2 and 10.5, respectively) in group 1 (oral contraceptive plus placebo), as shown in Table 6.

However, the influence of oral contraceptives on fibroadenoma is still questionable, considering that the studies in the literature are not uniform and the results are conflicting. Thus, some studies have demonstrated protective action through their use, for example the studies by Canny et al.^43^ and Rohan and Miller,^44^ while others found that oral contraceptives caused increased incidence of fibroadenoma, for example the study by Yu et al.^45^ We therefore put forward the theory that estriol, as a weak estrogen, may competitively block the stronger actions of the ethinyl estradiol present in oral contraceptives. This allows us to indirectly demonstrate that oral contraceptives have an important protective action in benign breast tumors, thereby resolving the doubts that persist in the literature.

Estriol is an estrogen that is weaker (by a factor of 1,000) than the ethinyl estradiol found in oral contraceptives, since it is a metabolic product.^41^ The possibility that selective antagonism might occur between it and other powerful estrogens like estradiol and estrone led us to use it in our study, in which it would be capable of competitively blocking the ethinyl estradiol found in oral contraceptives. We assumed that such antagonism would lead to a decrease in the protective effect of ethinyl estradiol on epithelium with fibroadenoma. The mechanism for estriol action works by competitive antagonism, i.e. by blocking estrogenic receptors and causing a smaller hormonal effect. Epithelium with fibroadenoma has these receptors in greater numbers than the stroma does.^40^ Thus, we expected a greater epithelial blockage caused by estriol than that was caused by stroma. There might be lower epithelial proliferation, which in turn would lead to decreased production of inhibition growth factors. Through the paracrine mechanism, the stroma would be less inhibited, which would then leave it free to proliferate, thereby obstructing the overall decrease in fibroadenomas.

**Figure 1:**
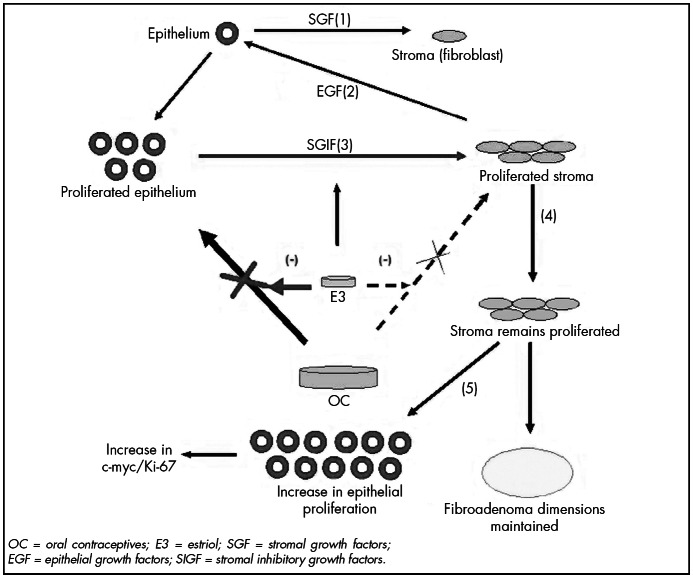
Model for paracrine control of fibroadenoma among users of oral contraceptive plus estriol. The sequential phases of the blockage that estriol caused to the protective effect of oral contraceptives on fibroadenoma are indicated by (1), (2), (3), (4) and (5).

On the other hand, proliferated stroma might not only maintain the tumor dimensions while the cycles evolve, but also increase the production of growth factors that would act on epithelium. This would lead to epithelial proliferation and create a tendency towards greater expression of the proteins relating to the cellular cycle (Ki-67 and c-myc). This was seen in group 2 (oral contraceptives plus estriol), in relation to group 1 (oral contraceptives plus placebo) (Figure 1).

Our immunohistochemical analysis on Ki-67 and c-myc expression did not show any statistically significant difference between group 1 (oral contraceptives plus placebo) and group 2 (oral contraceptives plus estriol). All we saw was a tendency towards greater expression of these proteins in the group 2 patients. We surmised that estriol blocked the ethinyl estradiol in the oral contraceptives, particularly in the epithelial compartment, thereby inducing lower proliferation of this compartment and leading to a lower production of stroma-inhibiting growth factors, which kept the stroma proliferated. In the end, the proliferated stroma produced growth factors that acted on epithelium to keep it in a higher proliferated condition, thereby demonstrating a tendency towards greater expression of proteins related to proliferation among the users of oral contraceptives plus estriol (group 2). This was demonstrated by the findings shown in Table 6, in which the results for Ki-67 and c-myc were 9.2 and 10.5 for group 1 (oral contraceptives plus placebo) and 10.8 and 17 for group 2 (oral contraceptives plus estriol), respectively. However, we did not find any statistical significance but only a tendency of behavior.

## CONCLUSION

The women who used hormonal oral contraception combined with estriol for four consecutive cycles showed Ki-67 and c-myc protein expression in epithelium with fibroadenoma that was similar to the expression shown by the women who used hormonal oral contraception with placebo for four cycles. Nevertheless, there was a greater tendency towards expression of these proteins among the users of oral contraceptives with estriol.
